# Attention‐deficit hyperactivity disorder symptoms and brain morphology: Addressing potential selection bias with inverse probability weighting

**DOI:** 10.1002/hbm.26562

**Published:** 2024-04-08

**Authors:** Annet Dijkzeul, Henning Tiemeier, Ryan L. Muetzel, Jeremy A. Labrecque

**Affiliations:** ^1^ Department of Child and Adolescent Psychiatry Erasmus MC University Medical Center Rotterdam‐Sophia Children's Hospital Rotterdam The Netherlands; ^2^ Department of Social and Behavioral Sciences Harvard T.H. Chan School of Public Health Boston Massachusetts USA; ^3^ Department of Radiology and Nuclear Medicine Erasmus MC University Medical Center Rotterdam Rotterdam The Netherlands; ^4^ Department of Epidemiology Erasmus MC University Medical Center Rotterdam Rotterdam The Netherlands

**Keywords:** attention problems, inverse probability weighting, population neuroscience, selection bias, structural MRI

## Abstract

The goal of this study was to examine what happens to established associations between attention deficit hyperactivity disorder (ADHD) symptoms and cortical surface and thickness regions once we apply inverse probability of censoring weighting (IPCW) to address potential selection bias. Moreover, we illustrate how different factors that predict participation contribute to potential selection bias. Participants were 9‐ to 11‐year‐old children from the Generation R study (*N* = 2707). Cortical area and thickness were measured with magnetic resonance imaging (MRI) and ADHD symptoms with the Child Behavior Checklist. We examined how associations between ADHD symptoms and brain morphology change when we weight our sample back to either follow‐up (ages 9–11), baseline (cohort at birth), or eligible (population of Rotterdam at time of recruitment). Weights were derived using IPCW or raking and missing predictors of participation used to estimate weights were imputed. Weighting analyses to baseline and eligible increased beta coefficients for the middle temporal gyrus surface area, as well as fusiform gyrus cortical thickness. Alternatively, the beta coefficient for the rostral anterior cingulate decreased. Removing one group of variables used for estimating weights resulted in the weighted regression coefficient moving closer to the unweighted regression coefficient. In addition, we found considerably different beta coefficients for most surface area regions and all thickness measures when we did not impute missing covariate data. Our findings highlight the importance of using inverse probability weighting (IPW) in the neuroimaging field, especially in the context of mental health‐related research. We found that including all variables related to exposure‐outcome in the IPW model and combining IPW with multiple imputations can help reduce bias. We encourage future psychiatric neuroimaging studies to define their target population, collect information on eligible but not included participants and use inverse probability of censoring weighting (IPCW) to reduce selection bias.

## Graphical abstract

Previously established associations between ADHD symptoms and structural MRI measures change substantially when potential selection bias is addressed. Estimated regression coefficients change up to 50%, with some getting stronger and others getting weaker. We provide recommendations for how neuroimaging studies can use inverse probability weighting to address potential selection bias.

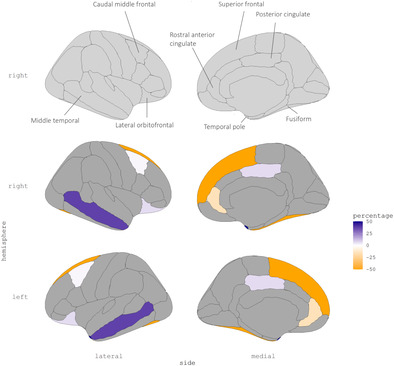

## 1 INTRODUCTION

Attention deficit hyperactivity disorder (ADHD) is among the most commonly diagnosed neuropsychiatric disorders in childhood. The disorder is characterized by age‐inappropriate levels of inattention and/or hyperactivity and can have a negative effect on multiple aspects of daily life for patients (American Psychiatric Association, 2013). Numerous neuroimaging studies have been conducted to examine possible structural brain alterations associated with ADHD; however, many of their findings have been inconsistent or even contradictory (Samea et al., 2019). While several explanations exist for these inconsistencies, various forms of bias have been proposed as a possible explanation for these inconsistent results, indeed adjusting for a more comprehensive set of confounding factors altered some associations between brain structure and ADHD symptoms (Dall'Aglio et al., 2022). Next to confounding bias, selection bias (e.g., self‐selection into a study or loss to follow‐up) is another possible source of bias that could alter the interpretation of results. Whether addressing selection bias could lead to more robust findings in the context of ADHD and the brain has not been explored before.

Selection bias occurs when reasons for participation or continuation in a study are related to the exposure and outcome under investigation (Hernán et al., 2004). Whether self‐selection or loss to follow‐up results in biased estimates depends on the specific relationship between exposure, outcome, and participation. For instance, in studies examining the association between child psychopathology and brain morphology, children of mothers with severe psychopathology might be more likely to drop out. Since this factor is also related to the child's psychopathology and brain morphology, the selection on this variable will likely induce selection bias. Depending on the characteristics which are over or under‐sampled, selection bias can result in either an underestimation or overestimation of the true effect.

Several studies have shown that participation or continuation in studies can depend on factors like sex, socio‐economic factors, health status, or psychopathology (Howe et al., 2013). Most of these factors are related to both ADHD and brain morphology (Russell et al., 2016). In addition, many neuroimaging studies are often performed in highly selected groups of individuals without a clearly defined target population. Therefore it is likely that neuroimaging studies of ADHD are susceptible to selection bias. Despite this, studies on the relation between ADHD symptoms and brain morphology generally do not address selection bias. The concern of selection bias can be illustrated from two observations in the literature, one neuroimaging study showed that changing sample composition based on socio‐economic status, ethnicity, and sex altered association between age and brain structure (LeWinn et al., 2017). Another study found that associations between several risk factors and ADHD were substantially different when corrected for possible selection bias (Biele et al., 2019). However, how selection bias potentially affects the specific relation between ADHD symptoms and brain morphology has not yet been investigated.

A potential solution and recommended strategy for addressing bias due to self‐selection or loss to follow‐up is inverse probability weighting (IPW) (Seaman & White, 2013). This technique generates weights as a function of the probability of ending up in the final sample used for analysis estimated using baseline information on participants, see Box 1 for a more in depth description of this method. The ability to examine and address potential selection bias depends largely on the information available at baseline. In this regard, prospective cohort studies offer a great potential to evaluate and address selection bias, as well as valuable insight into neurodevelopment in the context of ADHD. Specifically studies with a high baseline response, a well‐defined study base, and detailed baseline data on all participants recruited into the study enable identifying predictors of participation and are therefore important when it comes to addressing selection bias. In order to be able to explore selection bias as thorough as possible in a real‐world situation and identify many predictors of participation, this study was set in a prospective cohort study fulfilling the abovementioned criteria.

This study used data from a large population‐based cohort, the Generation R study, to try to address the issue of potential selection bias in ADHD and brain morphology. Specifically, the goal of this study was to examine what happens to previously established associations between ADHD symptoms and cortical surface and thickness regions once we apply inverse probability weighting. Moreover, we illustrate how different factors that predict participation contribute to potential selection bias.

## 2 MATERIALS AND METHODS

### 2.1 Study population

**FIGURE 1:**
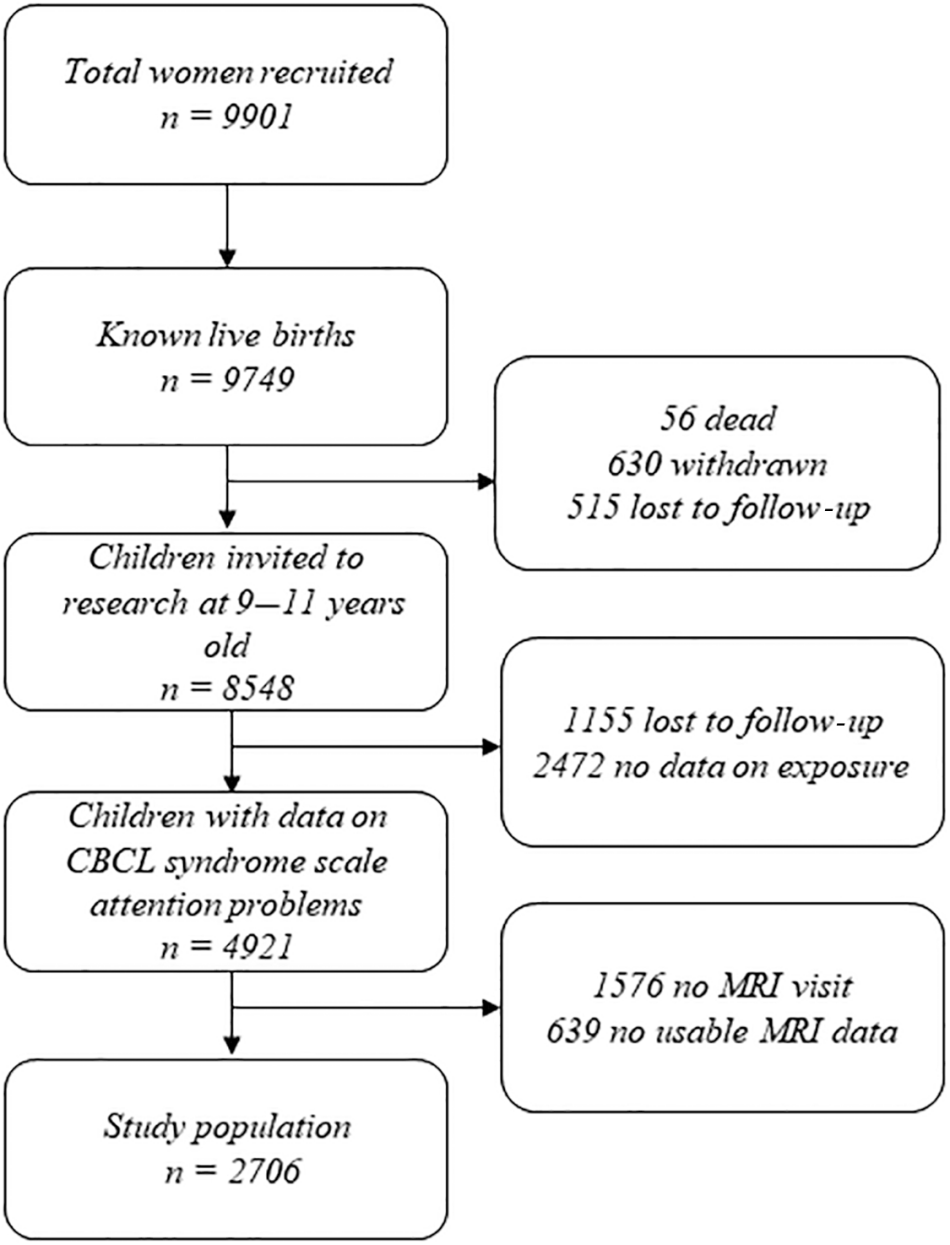
Flowchart of study participants in the Generation R cohort.

This study was embedded within the Generation R Study, a population‐based birth cohort in Rotterdam, the Netherlands, with data collection spanning from fetal life until early adulthood. A total of 9901 pregnant women living in Rotterdam with an expected delivery date between April 2002 and January 2006 were recruited, of which 9749 had a known live birth. Details of the study design have been described elsewhere (Kooijman et al., 2016). Parents provided written informed consent for themselves and their children. The Medical Ethics Committee of the Erasmus MC granted ethical approval for the study (METC‐20120165). In this study we used data from the baseline assessment (i.e., the prenatal assessment) and the assessment when the children were between 9 and 12 years old for creating weights. For the sample which was used for the association analysis, children with data on ADHD symptoms and T1‐weighted MRI images were included. Participants were excluded if they had incidental findings or if their brain scans failed processing or quality assurance procedures, this resulted in a study population of 2707 participants (see Figure 1).

### 2.2 Measures

#### 2.2.1 Attention problems

Children's attention problems, reported by the mother, were assessed using the Child Behavior Checklist CBCL (school‐age version) (Rescorla & Achenbach, 2001), a validated inventory widely used for parent reports of children's emotional and behavioral problems. The CBCL attention problems syndrome scale, an empirical scale consisting of 19 items scored on a three‐point Likert scale (0 = absent, 1 = occurs sometimes, 2 = occurs often), measures inattention, hyperactivity, and impulsivity and has previously been shown to have good discriminant validity and clinical utility in discriminating between children with and without ADHD diagnoses (Eiraldi et al., 2000).

#### 2.2.2 Image acquisition and processing

T1‐weighted MRI images were collected using a single, study‐dedicated MRI scanner (GE MR750W) with an eight‐channel receive‐only head coil. To acquire T1‐weighted structural images, a coronal inversion recovery fast spoiled gradient recalled (IR‐FSPGR) sequence was used (GE option BRAVO, TR = 8.77 ms, TE = 3.4 ms, TI = 600 ms, flip angle = 10°, matrix size = 220 × 220, field of view = 220 mm × 220 mm, slice thickness = 1 mm, number of slices = 230, ARC acceleration factor = 2). More details can be found elsewhere (White et al., 2018). Image processing was done using Freesurfer (version 6.0.0). Processing involved (i) removal of non‐brain tissue, (ii) correction of voxel intensities for B1 field inhomogeneities, (iii) tissue segmentation, and (iv) cortical surface‐based reconstruction. All images were visually inspected for inaccuracies in the surface‐based reconstruction by trained raters, as previously described in the literature (Muetzel et al., 2019). Poor quality reconstructions were excluded.

#### 2.2.3 Covariates

Variables included as predictors for inverse probability weighting were selected based on previous literature. Sex, age, gestational age, and birth weight were obtained through medical records at birth. Parental national origin (Dutch, European descent, Turkish/Moroccan, Surinames/Antillian, Other) was assessed based on the parents' birth country, in line with the Netherlands Central Office for Statistics (CBS). The following variables were collected by questionnaires during pregnancy: maternal age at enrolment (in years), maternal education level (primary education or lower, secondary education, university degree, or higher), maternal parity (nulliparous, one child, two, or more children), maternal smoking during pregnancy (never, smoking use until pregnancy known, continued smoking use during pregnancy), maternal alcohol consumption during pregnancy one or more glass/week for at least two trimesters (yes or no), marital status (married, living together, no partner), household net monthly income (low: <2000 euros, middle: 2000–3200 euros, high: >3200 euros). Maternal psychological distress was assessed using the Brief Symptom Inventory questionnaire global severity index (de Beurs, 2004). Whether the mother had ever experience depression anxiety or psychosis (yes or no) was assessed through questionnaires at pregnancy.

The exact variables used to create the different sets of weights can be found in Table S1. To investigate the contribution of different covariates to the IPW model, we fitted several models with each leaving a different group of covariates out. To minimize the number of models to be fitted we grouped similar covariates together. The first group of variables consisted of demographic variables relating to the child (sex and ethnicity). In the second group socioeconomic variables were included (maternal age at intake, maternal education, and household income). Maternal age at intake was included in this group because it can give additional information on socioeconomic status, as both education level and household income are related to this factor (e.g., women with higher education tend to have children at a later age). Remaining variables were grouped as family characteristics, substance use, child birth, psychopathology mother to allow us to explore the separate effect of these different types of (early) life exposure on selection bias. To see an overview of how variables were grouped see Table S2.

Variables included as confounders for the association analysis were the same as in the study by Hoogman et al. (2019), in order to reproduce this previous population‐based analysis. We included sex, age, and ethnic background as confounders. Intracranial volume was additionally included in the surface area analyses.

### 2.3 Statistical analyses

The R statistical software (version 4.1.2) was used for all analyses (R Core Team, 2022).

#### 2.3.1 Creating weights

BOX 1 **Inverse probability weighting**

Inverse probability weighting is a method that creates weights to address potential biases. It was initially developed to address selection bias in surveys, as proposed by Horvitz and Thompson (1952). The method has since then been adapted and widely used to address confounding bias as well, under the name inverse probability of treatment weighting (IPTW). In this study we focus on the method applied to address selection bias in studies with loss to follow‐up, known as inverse probability of censoring weighting (IPCW), also sometimes referred to as inverse probability of participation weighting, or inverse probability of attrition weighting. This method can be applied in studies that have enough detailed information of participants recruited into the study available at baseline. The first step is to select variables at baseline that are related to response as well as the exposure outcome under study, for instance socio‐economic factors or psychopathology. These variables are then used to estimate the probability of participation (or the probability of not being lost to follow‐up). The weights are subsequently created by taking the inverse of those probabilities, resulting in weights that give more weight to individuals with a low probability of participation compared to individuals with a high probability of participation. In this way, participants with a low probability of participation that nonetheless end up in the final analysis sample are given extra weight that helps them “represent” participants who were similar but that were actually lost to follow‐up. This creates a so‐called pseudo‐population that closely resembles the target population, for example, the sample at baseline.

Inverse probability of censoring weighting (IPCW) was used to adjust for potential selection bias due to loss to follow‐up, see Box 1. The weights were created by estimating the probability of not being lost to follow‐up using the *covariate balancing propensity score* R package (Imai & Ratkovic, 2014) and subsequently taking the inverse of those probabilities. Three types of weights were estimated, one set to weight the sample to the distribution of individuals participating in the follow‐up at 9–11 years old with CBCL data available. These weights were estimated by modeling the probability of being in the final analysis sample compared to being in the follow‐up sample conditional on the variables listed in the first column of Table S1.

The second set of weights was created to weight the analysis sample back to the cohort at birth, for example, baseline. These weights were estimated by modeling the probability of being in the final analysis sample compared to being in the cohort at birth conditional on the variables listed in the second column of Table S1. By taking the inverse of those probabilities we derived the respective weights. The covariates were balanced, based on the standardized mean differences.

The third and final set of weights was created to weight the sample to the distribution of the population of Rotterdam using publicly available data (CBS) on the distribution of characteristics in the Rotterdam population in 2002 till 2006, for example, the years in which the recruitment for Generation R took place. We used the distributions of *sex*, *ethnicity*, *maternal age at child birth*, *marital status*, and *parity*. Since individual level data was not available but only the population distributions were known, the weights had to be created through a raking procedure, using the *anesrake* R package (Pasek, 2018). Raking is a weighting method that generates weights for each individual participant based on the known population distributions. It is an iterative post‐stratification procedure to match the distributions of the analysis sample to the known population distributions. The procedures first multiply each individual by the inverse probability of having a certain characteristic based on the population distribution of that characteristic. Then the same is done for a second characteristic. This changes the matched distribution of the first characteristic, so then the first step is repeated. This process is repeated until there is convergence by which all of the weighted estimates match the population distributions. The raking procedure is particularly useful when only the marginal proportions for each variable separately are known and not the combination of variables (Kalton & Flores‐Cervantes, 2003).

#### 2.3.2 Multiple imputation

Because some individuals at baseline had partial non‐response we used multiple imputation to impute those missing values (Seaman & White, 2014). Missing data on baseline covariates were imputed with chained equations (25 imputed sets, 10 iterations) using the *mice* R package (van Buuren & Groothuis‐Oudshoorn, 2011). The weights were estimated in each imputed data set. The percentage of missing values was below 19% for all variables except for measures relating to psychopathology of the mother (psychological distress during pregnancy = 31.35%, depressed ever = 31.24%, anxious ever = 30.76%, psychoses ever = 30.8%).

#### 2.3.3 Outcome models

Multiple linear regressions were fitted to model the associations between attention problems and surface area and thickness in cortical regions. Analyses were adjusted for age at MRI scan, sex, ICV, and ethnicity, in order to replicate previous analysis by Hoogman et al. (2019). For the weighted regression robust standard errors using the *surveydesign* R package (Lumley, 2004) were calculated.

## 3 RESULTS

### 3.1 Sample characteristics

Characteristics of the Generation R cohort at different stages of follow‐up are displayed in Table 1, starting with the cohort at birth and ending with the study sample for which both MRI and CBCL data are available. Sample characteristics change when comparing the cohort at birth to the cohort at age 9–11 years old. Children for whom CBCL data is available at age 9–11 had a higher birth weight and higher gestational age. In addition, the members of the cohort at age 9–11 were more likely be of Dutch ethnicity, have higher maternal age at enrolment, higher maternal education, higher family income, lower in parity, less psychopathology, and more often without a partner. Although the change in sample characteristics is smaller when comparing the cohort at 9–11 with CBCL data to the final study sample, these trends track similarly. Differences between the final included and excluded samples are shown in Table S1.

**TABLE 1:** Population characteristics for different samples of Generation R cohort.

	Baseline (*n* = 9749)	Follow‐up (*n* = 4921)	Final sample (*n* = 2706)
Child characteristics			
Sex (girl)	4808 (49.3)	2484 (50.5)	1371 (50.7)
Gestational age at birth (months)	39.70 (2.01)	39.77 (1.89)	39.83 (1.82)
Birth weight (g)	3386.72 (582.83)	3417.84 (576.54)	3433.90 (564.18)
Ethnicity			
Dutch	4895 (53.8)	3188 (65.5)	1761 (65.7)
European descent	796 (8.8)	417 (8.6)	228 (8.5)
Turkish/Moroccan	1348 (14.8)	417 (8.6)	217 (8.1)
Surinames/Antillian	1064 (11.7)	420 (8.6)	226 (8.4)
Other	988 (10.9)	422 (8.7)	248 (9.3)
Maternal characteristics			
Age at enrolment (years)	29.94 (5.37)	31.38 (4.72)	31.44 (4.67)
Ethnicity			
Dutch	4575 (50.5)	3090 (63.6)	1681 (62.7)
European descent	764 (8.4)	410 (8.4)	226 (8.4)
Turkish/Moroccan	1399 (15.4)	412 (8.5)	221 (8.2)
Surinames/Antillian	1097 (12.1)	395 (8.1)	219 (8.2)
Other	1233 (13.6)	555 (11.4)	333 (12.4)
Education during pregnancy			
Primary	950 (11.1)	238 (5.1)	114 (4.5)
Secondary	3920 (45.9)	1850 (40.0)	978 (38.5)
Higher	3666 (42.9)	2540 (54.9)	1447 (57.0)
Parity			
0	5168 (55.1)	2797 (58.7)	1570 (60.1)
1	2837 (30.2)	1418 (29.8)	766 (29.3)
≥2	1381 (14.7)	550 (11.5)	278 (10.6)
Continued smoking during pregnancy	1483 (18.0)	580 (13.3)	293 (12.2)
Alcohol (1 or more glass/week for at least 2 trimesters) during pregnancy (yes vs. no)	576 (7.3)	412 (9.9)	228 (10.1)
Addiction (yes vs. no)	204 (3.0)	102 (2.7)	52 (2.4)
Psychological distress score during pregnancy^a^	0.30 (0.38)	0.25 (0.32)	0.24 (0.31)
Depression ever (yes vs. no)	1825 (27.6)	1064 (28.4)	595 (28.5)
Anxiety ever (yes vs. no)	973 (14.5)	509 (13.4)	270 (12.8)
Psychosis ever (yes vs. no)	113 (1.7)	51 (1.3)	20 (0.9)
Household characteristics			
Marital status mother			
Married	4256 (49.8)	2326 (50.5)	1264 (50.1)
Living together	3069 (35.9)	1834 (39.8)	1018 (40.4)
No partner	1220 (14.3)	446 (9.7)	239 (9.5)
Monthly income during pregnancy (>2200 euros)	3684 (54.5)	2611 (66.0)	1476 (68.1)

*Note*: Values are *n* (percentages) for categorical, mean (standard deviation) for continuous variables. Baseline: cohort at birth. Follow‐up: sample at age 9–11 years old with CBCL data available. Final sample: sample of participant with both CBCL and MRI data.

^a^ Score ranging from 0 to 4.

### 3.2 Inverse probability weighting

**FIGURE 2:**
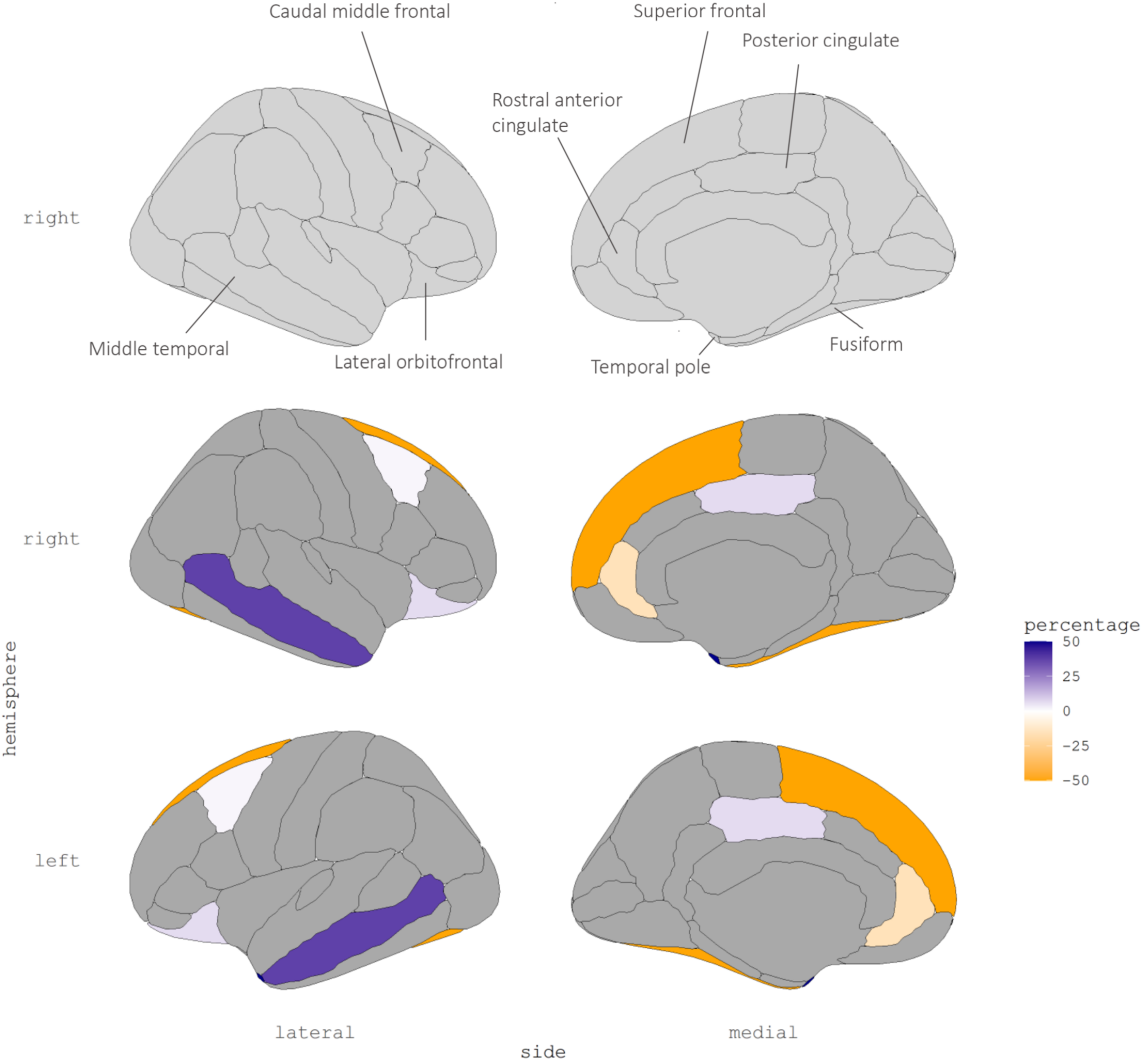
Percentage change in estimated regression coefficients for the CBCL attention problems scale per cortical region after adding weights to the linear models to weight analysis sample back to cohort at birth.

We analyzed surface area and thickness for all regions, but focused specifically on regions identified as being ADHD‐associated cortical features in a previous mega‐analysis from the ENIGMA‐ADHD Working group (Hoogman et al., 2019). Adjustment using IPCW to weight back to the follow‐up cohort (age 9–11 years old) with data on the CBCL assessment did not lead to substantial change in the beta coefficients. However, weighting to baseline (cohort at birth) resulted in both increases and decreases in beta coefficients (Figure 2). Subsequently weighting to the eligible population (Rotterdam) resulted in more or less similar changes in beta coefficients as weighting to the cohort at birth for most surface area and thickness measures (Table 2).

**TABLE 2:** Unweighted and weighted estimated association between cortical regions and CBCL syndrome scale attention problems.

Cortical regions	*B*	SE	Beta	CI lower	CI upper	*p*	% change
Surface areas							
Caudal middle frontal gyrus							
Participated (unweighted)	−13.70	5.50	−0.037	−24.49	−2.91	.01	
IPW to follow‐up	−14.30	5.51	−0.039	−25.10	−3.49	.01	4.38
IPW to baseline	−13.93	6.60	−0.037	−26.87	−0.99	.03	1.68
IPW to eligible (Rotterdam)	−13.87	6.90	−0.036	−27.41	−0.33	.04	1.24
Lateral orbitofrontal cortex							
Participated (unweighted)	−8.67	5.02	−0.025	−18.52	1.17	.08	
IPW to follow‐up	−8.82	5.16	−0.025	−18.92	1.29	.09	1.73
IPW to baseline	−9.22	5.79	−0.027	−20.58	2.14	.11	6.34
IPW to eligible (Rotterdam)	−8.59	6.01	−0.025	−20.37	3.20	.15	−0.92
Middle temporal gyrus							
Participated (unweighted)	−13.86	5.87	−0.031	−25.37	−2.35	.02	
IPW to follow‐up	−14.81	6.01	−0.033	−26.59	−3.03	.01	6.58
IPW to baseline	−19.05	7.40	−0.043	−33.56	−4.54	.01	37.45
IPW to eligible (Rotterdam)	−18.74	7.86	−0.042	−34.15	−3.33	.02	35.21
Posterior cingulate cortex							
Participated (unweighted)	−4.65	2.42	−0.028	−9.40	0.09	.05	
IPW to follow‐up	−4.90	2.44	−0.029	−9.68	−0.12	.04	5.38
IPW to baseline	−4.95	2.71	−0.029	−10.27	0.36	.07	6.45
IPW to eligible (Rotterdam)	−4.36	2.84	−0.026	−9.92	1.20	.12	−6.24
Rostral anterior cingulate cortex							
Participated (unweighted)	−3.58	1.94	−0.026	−7.38	0.22	.06	
IPW to follow‐up	−3.52	1.98	−0.025	−7.41	0.36	.08	−1.68
IPW to baseline	−3.02	2.28	−0.022	−7.49	1.46	.19	−15.64
IPW to eligible (Rotterdam)	−2.76	2.42	−0.020	−7.49	1.98	.25	−22.91
Superior frontal gyrus							
Participated (unweighted)	−7.10	11.97	−0.007	−30.56	16.37	.55	
IPW to follow‐up	−7.48	11.84	−0.007	−30.69	15.73	.53	5.35
IPW to baseline	−3.53	13.57	−0.002	−30.12	23.07	.79	−50.28
IPW to eligible (Rotterdam)	−11.89	13.66	−0.012	−38.66	14.88	.38	67.46
Total surface area							
Participated (unweighted)	−320.46	77.38	−0.036	−472.18	−168.73	<.001	
IPW to follow‐up	−335.89	78.75	−0.037	−490.24	−181.55	<.001	4.81
IPW to baseline	−320.30	89.70	−0.036	−496.11	−144.48	<.001	−0.05
IPW to eligible (Rotterdam)	−315.02	92.83	−0.035	−496.98	−133.07	<.001	−1.70
Thickness							
Fusiform gyrus							
Participated (unweighted)	0.004	0.002	0.040	0.000	0.008	.04	
IPW to follow‐up	0.004	0.002	0.042	0.000	0.008	.03	0.04
IPW to baseline	0.006	0.003	0.059	0.001	0.011	.02	45.28
IPW to eligible (Rotterdam)	0.006	0.003	0.060	0.001	0.011	.02	50.16
Temporal pole							
Participated (unweighted)	0.012	0.007	0.034	−0.002	0.025	.08	0.084
IPW to follow‐up	0.011	0.007	0.032	−0.002	0.025	.10	−8.33
IPW to baseline	0.006	0.008	0.018	−0.009	0.022	.43	−50.00
IPW to eligible (Rotterdam)	0.007	0.008	0.021	−0.009	0.024	.36	−35.74

*Note*: Regions are average of left and right hemisphere surface area. Model adjusted for age, sex, and ethnic background. ICV included as covariate in the surface area analysis.

Abbreviations: *B*, unstandardized regression coefficient for the square root transformed CBCL syndrome scale attention problem score; beta, standardized regression coefficient; CI, 95% confidence interval of that regression coefficient; IPW, inverse probability weighted; % change, percentage change in regression coefficient compared to unweighted regression coefficient.

Two of the previously established associations with attention problems became stronger after weighting. We observed that the unweighted beta coefficient for the middle temporal gyrus surface area (−13.86, 95% CI [−25.37, −2.35]) increased in magnitude by 6.6% when weighting to the follow‐up cohort (−14.81, 95% CI [−26.59, −3.03]) and increased in magnitude by 37.5% when weighting to baseline (−19.05, 95% CI [−33.56, −4.54]). The coefficients weighted to the population of Rotterdam at time of recruitment remained similar to the coefficient weighted to baseline. A similar effect was observed for the fusiform gyrus thickness. The unweighted beta coefficient (0.004, 95% CI [0.000, 0.008]) did not change when weighting to follow‐up, though we observe an increase of 45.28% when weighting to baseline (0.006, 95% CI [0.001, 0.011]), and an additional increase of 5% when weighed to the Rotterdam population.

For the rostral anterior cingulate surface area we observed a decrease in the association with attention problems after weighting. The beta coefficient did not change when weighting to follow‐up. However, compared to the unweighted coefficients (−3.58, 95% CI [−7.38, 0.22]), weighting to baseline decreased beta coefficients with 15.64% (−3.02, 95% CI [−7.49, 1.46]) and weighting to eligible resulted in a decrease of 22.91% (−2.67, 95% CI [−7.49, 1.98]).

### 3.3 Role of different predictor variables in estimating weights

For two of the cortical measures (middle temporal gyrus surface area and temporal pole thickness) most affected by the weighting we tested different models to disentangle to which extent different variables contribute to the changes in the weighted regression coefficients. For both measures, we observed that removing one group of variables resulted in the weighted regression coefficient moving closer to the unweighted regression coefficient. Removing variables relating to socio‐economic factors and demographics seems to have the greatest apparent impact on changes in coefficients for both the middle temporal gyrus surface area as well as the temporal pole thickness (Table 3).

**TABLE 3:** Weighted estimated association between cortical regions and CBCL syndrome scale attention problems obtained by different models.

Cortical region	IPW method—response model	*B*	SE	Beta	CI lower	CI upper	*p*
Surface area							
Middle temporal gyrus	Full model	−19.05	7.40	−0.043	−33.56	−4.54	.01
	Without demographics	−17.63	7.50	−0.039	−32.33	−2.92	.02
	Without SES	−17.47	6.66	−0.040	−30.52	−4.42	.01
	Without family characteristics	−18.42	7.32	−0.041	−32.77	−4.07	.01
	Without substance use	−18.11	7.35	−0.040	−32.52	−3.69	.01
	Without child birth	−18.30	7.39	−0.041	−32.79	−3.82	.01
	Without psychopathology	−18.47	7.20	−0.041	−32.58	−4.36	.01
Thickness							
Temporal pole	Full model	0.006	0.008	0.018	−0.009	0.022	.43
	Without demographics	0.009	0.008	0.026	−0.007	0.025	.25
	Without SES	0.011	0.008	0.031	−0.004	0.026	.16
	Without family characteristics	0.008	0.008	0.023	−0.008	0.024	.31
	Without substance use	0.008	0.008	0.024	−0.007	0.024	.30
	Without child birth	0.008	0.008	0.022	−0.007	0.023	.31
	Without psychopathology	0.008	0.008	0.021	−0.008	0.023	.33

*Note*: Regions are average of left and right hemisphere surface area. Models adjusted for age, sex, and ethnic background. ICV included as covariate in the surface area analysis.

Abbreviations: *B*, unstandardized regression coefficient for the square root transformed CBCL syndrome scale attention problem score; beta, standardized regression coefficient; CI, 95% confidence interval of that regression coefficient; IPW, inverse probability weighted.

### 3.4 Effect of using multiple imputation for missing predictor variables

Because of missingness in the predictors of inclusion (i.e., the variables used to create the weights), we used multiple imputation to impute these missing values (see Section 2). To investigate the effect of imputing missing values in the predictor variables, we reran analysis for weighting back to the cohort at birth without imputing these predictors, resulting in an analysis among participants with complete covariates. As a consequence, less observations were available for analysis; *n* = 1773 instead of *n* = 2701. This is because only the observations for which we were able to create weights, for example, those who had no missingness in the predictors of inclusion, could be used in the weighted analysis. Those analyses showed considerably different beta coefficients for most surface area regions and all thickness measures (Table 4). Some beta coefficients were more similar to the unweighted analyses while others changed in the opposite direction.

**TABLE 4:** Estimated association between cortical regions and CBCL syndrome scale attention problems weighted back to cohort at birth using imputed data (*n* = 9749) or data with only complete cases (*n* = 4485).

Cortical region		*B*	SE	CI lower	CI upper	*p*
Surface area						
Caudal middle frontal gyrus	Unweighted	−13.70	5.50	−24.49	−2.91	.01
	IPCW + MI	−13.93	6.60	−26.87	−0.99	.03
	IPCW only	−16.87	7.40	−31.39	−2.35	.02
Lateral orbitofrontal cortex	Unweighted	−8.67	5.02	−18.52	1.17	.08
	IPCW + MI	−9.22	5.79	−20.58	2.14	.11
	IPCW only	−1.99	7.03	−15.78	11.80	.78
Middle temporal gyrus	Unweighted	−13.86	5.87	−25.37	−2.35	.02
	IPCW + MI	−19.05	7.40	−33.56	−4.54	.01
	IPCW only	−13.82	8.42	−30.32	2.69	.10
Posterior cingulate cortex	Unweighted	−4.65	2.42	−9.40	0.09	.05
	IPCW + MI	−4.95	2.71	−10.27	0.36	.07
	IPCW only	−4.19	3.22	−10.51	2.12	.19
Rostral anterior cingulate cortex	Unweighted	−3.58	1.94	−7.38	0.22	.06
	IPCW + MI	−3.02	2.28	−7.49	1.46	.19
	IPCW only	−3.57	2.60	−8.68	1.53	.17
Superior frontal gyrus	Unweighted	−7.10	11.97	−30.56	16.37	.55
	IPCW + MI	−3.53	13.57	−30.12	23.07	.79
	IPCW only	−7.32	15.48	−37.68	23.04	.64
Total surface area	Unweighted	−320.46	77.38	−472.18	−168.73	<.001
	IPCW + MI	−320.30	89.70	−496.11	−144.48	<.001
	IPCW only	−299.23	113.27	−521.37	−77.10	.01
Thickness						
Fusiform gyrus	Unweighted	0.004	0.002	0.000	0.008	.04
	IPCW + MI	0.006	0.008	−0.009	0.022	.43
	IPCW only	0.005	0.009	−0.014	0.023	.61
Temporal pole	Unweighted	0.012	0.007	−0.002	0.025	.08
	IPCW + MI	0.006	0.003	0.001	0.011	.02
	IPCW only	0.004	0.003	−0.001	0.010	.14

*Note*: Regions are the average of left and right hemisphere surface area. Model is adjusted for age, sex, and ethnic background. ICV is also included as a covariate in the surface area analysis.

Abbreviations: *B*, unstandardized regression coefficient for the square root transformed CBCL syndrome scale attention problem score; CI, 95% confidence interval of that regression coefficient; IPCW, inverse probability of censoring weighting; MI, multiple imputation.

## 4 DISCUSSION

In this study, we applied inverse probability weighting to address potential selection bias in the association between ADHD symptoms and brain morphology. We showed that associations between brain structure and ADHD symptoms changed, in some cases substantially, when weighting our sample to baseline or the population of Rotterdam.

### 4.1 Estimates obtained from weighted analyses differ from unweighted

We found that most associations changed after weighting our analysis using inverse probability weights, some of the associations between ADHD symptoms and cortical regions were stronger while others were weaker. Specifically, we found that the association between ADHD symptoms and the surface area of the middle temporal gyrus, as well as the thickness of the fusiform gyrus, were stronger after weighting. Previous attempts in population studies did not found significant associations of thickness measures with ADHD symptoms (Dall'Aglio et al., 2022), while other studies do find these associations with ADHD (Shaw et al., 2007). Selection bias could potentially be a part of the explanation for this discrepancy. The association of the rostral anterior cingulate cortex with ADHD symptoms substantially reduced after weighting. Few studies have implicated structural differences in the cingulate cortex, the ones that do might have overestimated this association due to selection bias. However, comparisons to previous findings should be made with caution due to other factors relating to study design or methods, besides selection bias, that could explain some of the differences. Finally, the association of ADHD symptoms with total surface area was much less affected by weighting compared to more focused regions of interest, suggesting this association is less susceptible to selection bias. Our results do show that factors predicting participation and attrition, like the ones considered in this study (i.e., socio‐economic status, psychopathology, and health factors), can bias the relation between ADHD symptoms and specific regions of the brain in both directions.

### 4.2 Specification of the response model

Next we explored how different groups of variables contribute to selection bias. The main model used to correct for selection bias included variables relating to demographics, socio‐economic status, family characteristics, substance use, child birth, and psychopathology of the mother. We suspected all of these variables were possibly causing selection bias after identifying that those could be both related to participation as well as ADHD and brain morphology. By testing several models, each time leaving one group of variables out, we found that each group of variables were important to include. However, results changed most when removing variables relating to socio‐economic status (SES), indicating that those variables appeared to contribute most to the selection bias. Accordingly, we observed that in our final sample more people with low SES were lost to follow‐up, meanwhile lower SES is associated with both ADHD and variation in brain structure (Noble et al., 2015; Russell et al., 2016). Thus, the occurrence of selection bias due to SES factors is very likely in this case, explaining the stronger shift in effect estimates and corresponding significance when removing these variables from the model. Little literature exists pertaining to which type of variables should be included into the response model used for creating the weights. Typically in studies that apply IPCW, it is common practice to build the response and outcome model independently. Here the main focus is more on fitting a model that perfectly predicts response and not to optimize the response model in relation to the outcome model, thus leaving variables out if they do not “significantly” improve prediction. Hernán et al. (2004) suggested it is important to include all predictors of both response and outcome. In addition, Seaman and White (2013) recommended excluding variables that are only related to response and not to the outcome or exposure, but including variables associated with both exposure and outcome or solely with the outcome. In this context, variables should not be removed solely based on how well they contribute to the prediction model. In support of these recommendations, we found that leaving covariates related to both response and the exposure/outcome out of the model increases bias. For this reason, we recommend building the response model based on DAGs and previous knowledge and to include all variables that are related to both response and the exposure outcome relation under study.

### 4.3 Combining inverse probability of censoring weighting with multiple imputation

We also assessed the impact of not imputing missing data in the covariates used for building the weights. As in many studies, we found that there was some missing covariate data at baseline due to partial non‐response (e.g., questions not filled in, failed measurements). The baseline data is the data that was used to create weights. Most data (around 30%) were missing on maternal psychopathology; we defined this variable as an important one to include in the model. We hypothesized that a mother's mental health can have an effect on the child's psychopathology and brain morphology while also potentially influencing participation/continuation in the study. Indeed in later stages of the study, we observe less participants with mothers with psychopathology. Disregarding the issue of missing data would mean that we could either not use maternal psychopathology as a variable in the model or only create weights for participants with data on maternal psychopathology, which would again create a selected sample which was possibly biased. We investigated the impact of the latter by not imputing data. Our results showed that when weights were estimated based on non‐imputed data this indeed also resulted in biased estimates, showing that the combination of both methods is crucial when there is missing data in the variables used to create weights. These findings further illustrate what has been described previously in the context of combining multiple imputations with IPTW (Seaman et al., 2012). Imputation methods are mainly useful in scenarios of partial non‐response as there is enough auxiliary information on which the imputation can be based. In the case of loss to follow‐up, for example, complete non response, there is a high risk of misspecification of the imputation model (Seaman et al., 2012). This is where inverse probability weighting is most useful. Therefore, we argue that it is most beneficial to combine both MI and IPW when there is loss to follow‐up and partial missing covariate data at baseline.

### 4.4 The advantage of rich baseline data

In general, our results provide more evidence for the presence of bias due to loss to follow‐up in comparison to several previous investigations of selection bias in large prospective cohort studies (Bliddal et al., 2018; Greene et al., 2011; Nohr & Liew, 2018; Wolke et al., 2009). This can be explained in several ways. First, previous studies did not focus specifically on neuroimaging in the context of ADHD. This particular association is perhaps more susceptible to selection bias then others, for instance due to unorganized behavior in parents and children causing them to be less likely to successfully continue participation in studies. Second, selection bias and missing data can be addressed in a number of ways, and as our results demonstrated this could lead to different results. Less information, either due to missing data or by leaving out variables, will affect how well the weights perform. The Generation R cohort offered the advantage of including many relevant variables to create the response model for weighting to baseline. However, additionally weighting to the Rotterdam population resulted in a much smaller change in estimates, even though we know our baseline sample differs from the target population (Jaddoe et al., 2012). This could be due to the fact that we had less information to weight our sample to the Rotterdam population then weighting to baseline (i.e., several factors which related to attrition were not part of the weights); however, it could also mean Generation R generally has a good baseline response rate. A characteristic of the Generation R cohort is the relatively high baseline response (62%) compared to other studies. It may be that, a less motivated or more disadvantaged population is initially recruited which may lead to higher subsequent loss to follow‐up. As a consequence of these higher loss to follow‐up, weighting can have more of an effect than it would have in studies with a more selected or motivated baseline sample. However, in studies with a more selected baseline sample there could be more bias due to selection mechanisms that are related to baseline recruitment. Often those selection mechanisms are unknown and this complicates the generalizability to a target population (Jöckel & Stang, 2013; Westreich et al., 2019). Thus, although a high baseline response may result in higher loss to follow‐up and potentially more selection bias, with sufficient information on non‐participants, it creates the possibility to apply weights that bring estimates closer to the true effect in the target population.

### 4.5 Application to other types of psychiatric neuroimaging studies

The abovementioned considerations do apply to the broader psychiatric neuroimaging field. In clinical studies, often utilizing a case control design, there should be more consideration for factors inducing selection bias and threats to generalizability. Although smaller clinically focused studies might have less loss to follow‐up compared to large cohort studies, there are still several mechanisms through which selection bias can occur. For instance, excluding scans after quality control could already induce selection bias, as someone with ADHD might have more difficulty with laying still in the scanner and thus has a higher chance of being excluded due to a poor quality scan. In addition, most studies rely on highly selective study samples that are not representative of a well‐defined target population. Often these studies are designed without even defining a target population, for example, the specific group or population to which the study findings are intended to be generalized or applied. The study base, the population from which the study participants are selected, is often far from representative of a reference pool due to convenience sampling, the choice to include only extreme cases or selection of “well” controls (Schwartz & Susser, 2011). This induces another potential source of selection bias due to unknown selection mechanisms related to baseline recruitment as illustrated in the previous paragraph. However, selection bias is rarely addressed within neuroimaging studies. Our findings demonstrate that this could lead to a biased interpretation of results. Thus, in order to address these issues, future psychiatric neuroimaging studies should first of all be clear in defining a target population to which they want to generalize their results to. Secondly, future studies should try to obtain sufficient information on eligible people from the target population that do not end up in the final analysis sample. This allows researchers to at least be aware of the extent of too which selection bias might be present. Third, we encourage researchers to address selection bias using inverse probability weighting in order to get more robust results.

### 4.6 Limitations

A few limitations must be kept in mind when interpreting our results. While inverse probability weighting addresses selection bias due to variables included in the response model, there might be unmeasured factors related to participation and outcome not included in our model. However, the aim of our study was to illustrate the use of inverse probability weighting not to explore the full magnitude of potential bias. Additionally, our results are restricted to neuroimaging in the context of ADHD. However, it is likely that these results also generalize to other types of psychiatric neuroimaging studies, since many of the factors that we included to address selection bias not only relate to ADHD but also to other types of psychiatric disorders. Third, this study relied solely on symptom‐level data for ADHD within the general population. Nevertheless, the brain alteration explored in this study has also been linked to individuals with an ADHD diagnoses in a large consortium of 36 centers (ENIGMA‐ADHD Working group). The brain differences that were found between individuals with and without ADHD diagnoses also became apparent when performing a continuous analysis to find brain features associated with ADHD symptoms within the general population (Hoogman et al., 2019). These findings suggest that not only ADHD symptoms but also brain phenotypes lie on a continuum. Therefore we suspect our results could also have implications for studies with clinical samples. However, whether the impact of selection bias would be more or less profound in clinical samples is still unclear. To improve the generalizability of our findings beyond the population‐based study, we suggest future research to investigate the impact of selection bias on associations between brain structure and ADHD in clinical samples.

## 5 CONCLUSION

To conclude, in this study, IPCW was used to address selection bias in a cohort study with a high baseline response. We found that associations between ADHD symptoms and brain structure were altered, both stronger and weaker, when using inverse probability weighting. In addition, we showed that leaving out variables identified as predictors of participation that are related to exposure‐outcome resulted in more bias. Finally, we demonstrated the importance of combining IPCW with MI when there is missing data in variables identified as predictors of participation. Taken together, the findings from this study suggest that non‐random selection mechanisms within neuroimaging studies can lead to biased results. To prevent systematic biased interpretation of results we encourage future psychiatric neuroimaging studies to (i) clearly define a target population, (ii) try to obtain and use information on eligible participants that are not included in the final analysis sample, and (iii) use inverse probability of censoring weighting. Our results demonstrate how inverse probability weighting can be used and highlight the importance of doing so in the neuroimaging field, especially in the context of mental health related topics.

## FUNDING INFORMATION

High performance computing for image analysis was provided by the Dutch Organization for Scientific Research (NWO 2021.042, “Snellius” [http://www.surf.nl]); Erasmus MC Fellowship; NWO‐ZonMW: 016.VICI.170.200; NWO‐ZonMW Veni: 09150162010213; Stichting Vrienden van het Sophia: WAR21‐72.

## Supporting information


**Table S1.** Variables used in logistic regression model to calculate inverse probability of attrition weights.
**Table S2.** Grouped variables.

## Data Availability

The data that support the findings of this study are not publicly available due to privacy and ethical restrictions. Reasonable requests to access the datasets should be directed to the Director of the Generation R Study, Vincent Jaddoe (generationr@erasmusmc.nl), in accordance with the local, national, and European Union regulations.
