# Supplementary material for: Attention‐deficit hyperactivity disorder symptoms and brain morphology: Addressing potential selection bias with inverse probability weighting
Source: Hum Brain Mapp. 2024 Apr 8;45(5):e26562. doi: 10.1002/hbm.26562 (PMC11002333; doi:10.1002/hbm.26562)
Supplement: Supplementary file 1 — Table S1. Variables used in logistic regression model to calculate inverse probability of attrition weights. Table S2. Grouped variables. [file HBM-45-e26562-s001.docx]

**SUPPLEMENTARY TABLES**

| Table S1 Variables used in logistic regression model to calculate inverse probability of attrition weights | |
| --- | --- |
| Follow-up (CBCL at age 9-11) | Baseline (cohort at birth) |
| Sex | Sex |
| Gestational age at birth | Gestational age at birth |
| Birth weight | Birth weight |
| Ethnicity child | Ethnicity child |
| Age mother at intake | Age mother at intake |
| Maternal education | Maternal education |
| Parity | Parity |
| Smoking during pregnancy | Smoking during pregnancy |
| Alcohol during pregnancy | Alcohol during pregnancy |
| Monthly household income | Monthly household income |
| Marital status | Marital status |
| Psychopathology mother (reported during pregnancy) | Psychopathology mother (reported during pregnancy) |
| - Psychological distress during pregnancy | - Psychological distress during pregnancy |
| - Depression (ever) | - Depression (ever) |
| - Anxious (ever) | - Anxious (ever) |
| - Psychosis (ever) | - Psychosis (ever) |
| Child problems at age 9-11 year |  |
| - CBCL Total problems score |  |
| - CBCL DSM oriented scale ADHD problems |  |

| Table S2 Grouped variables​ | |
| --- | --- |
| Group​ | Variables​ |
| Demographics ​ | Sex​ |
| ​ | Ethnicity child​ |
| ​ | ​ |
| Socio-economic status​ | Age mother at intake​ |
| ​ | Maternal education​ |
| ​ | Monthly household income​ |
| ​ | ​ |
| Family characteristics​ | Parity​ |
| ​ | Marital status​ |
| ​ | ​ |
| Substance use​ | Smoking during pregnancy​ |
| ​ | Alcohol during pregnancy​ |
| ​ | ​ |
| Child birth​ | Gestational age​ |
| ​ | Birth weight​ |
| ​ | ​ |
| Psychopathology mother​ | Psychological distress during pregnancy​ |
| ​ | Depression (ever)​ |
| ​ | Anxious (ever)​ |
| ​ | Psychosis (ever)​ |
